# Supplementary material for: Cross- analyzing the opinions and experiences of nurses, physiotherapists, dentists, midwives, and pharmacists with respect to addictive disorder screening in primary care: A qualitative study
Source: PLoS One. 2025 Jan 24;20(1):e0315926. doi: 10.1371/journal.pone.0315926 (PMC11759999; doi:10.1371/journal.pone.0315926)
Supplement: S1 Appendix — (DOCX) [file pone.0315926.s001.docx]

Realities of identifying substance-related and addictive disorders in primary care
 primary care: detection by healthcare professionals

*My name is Agathe Edeline, or Amélie Tripault, and I'm a doctoral student in general medicine in Tours. The subject of my thesis is the realities of identifying problem behaviors and addictions. It is part of a wider project, PAPRICA (Problematic use and Addictions in Primary Care), which aims to identify the problems encountered in identifying problematic use and addictions, and to address them.*
*problematic use of drugs and to find solutions.*

*This is a qualitative study based on interviews with healthcare professionals (physiotherapists, nurses, midwives, pharmacists and dentists) in the Centre, Normandie and Île-France french regions.*
*femme, pharmacien et dentiste) in the Centre, Normandie and Île-de-France regions.*

*The interview is recorded anonymously. It will then be transcribed in writing and analyzed using the grounded theory method.*

Icebreaker questions :

- Explain to me the structure in which you work.

- Tell me the story of the last patient with an addiction problem you saw?

- Tell me the story of a patient where the problem of addiction was not identified early enough to avoid complications.

Or the story of a scouting trip that went well.

Do you think you have a role to play in screening for addictions?

-Why do you think someone confided in you about their addictions? What's the bonus of your profession?

Which referrers/health professionals do you refer to?

-What is your relationship with general practitioners in the field of addiction?

-Do you work with addictologists?

What obstacles do you see to locating?

What improvements do you think are possible for screening?
Finally: Do you have anything to add on this subject?

| Gender |  |
| --- | --- |
| Age |  |
| Exercise area |  |
| Exercise mode |  |
| Specialty, training, ... |  |
