# Supplementary material for: Cross- analyzing the opinions and experiences of nurses, physiotherapists, dentists, midwives, and pharmacists with respect to addictive disorder screening in primary care: A qualitative study
Source: PLoS One. 2025 Jan 24;20(1):e0315926. doi: 10.1371/journal.pone.0315926 (PMC11759999; doi:10.1371/journal.pone.0315926)
Supplement: Attachment — Submitted filename: PLOS ONE - Reviewer feedback.docx [file pone.0315926.s003.docx]

Thank you for the opportunity to review this article. I found it to be an interesting read and believe that there is not a lot of research done in this important area. However, I would suggest the following minor and major edits to improve the piece:

**Data availability:**

It is recommended that authors include selected verbatim interview quotations and focus group data underlying the findings.

**Abstract:**

This is well written and gives a nice overview of the paper!

Consider the following minor points for improvement:

1. Consider adding another 1-2 keywords.
2. The spelling of the word ´analysing´ is spelt differently in the full and short title. Authors should ensure this is spelt similarly throughout the paper.
3. Consider combining the background and objective section of the abstract. Consider adding the main aim(s) of the research.
4. Correct the spelling error of the word between in the methods section of the abstract:

*“Methods: This qualitative study inspired by the grounded theory was carried out betwenn August 2018 and July 2019.”*

**Introduction:**

Consider the following major and minor points for improvement:

The introduction is well written. However, I found it very short and lacking relevant information. There are also several grammatical, spelling, and punctuation errors throughout which I would advise authors to correct as well as the rest of the paper.

5. Punctuation is missing throughout the introduction. In the first sentence of the first paragraph of the introduction, add a comma after the word mortality, and in the second sentence, add a comma after the word opioids. Second paragraph of the introduction, add a comma after the word inefficiency and after the word midwives in both sentence 3 and sentence 7. Correct the spelling of the word `midwifes´. The authors should check for further missing punctuation, spelling and grammar errors throughout the manuscript.

*“The global burden of addiction disorders is based on their morbidity, mortality and social costs1,2. Alcohol, opioids and cannabis are the most prevalent, with a risk factor for premature death and disability3–5.”*

*“But many already known obstacles to addictive disorder screening in primary care remain, such as lack of time, a feeling of inefficiency and patient reluctance14–19.”*

*“Since 2016, dental surgeons, nurses, midwifes and physiotherapists have been called upon to carry out this screening21(p201).”*

*“Since 2016, dental surgeons, nurses, midwifes and physiotherapists have been called upon to carry out this screening21(p201).”*

6. Authors could give examples or add to the sentence in the first paragraph of the introduction to make readers more familiar with the DSM-V criteria:

“*The fifth edition of the DSM-V described the criteria for diagnosing dependence for both drug related disorders and non-drug-related behaviours7.*”

7. Authors should extend/give a brief explanation to readers as to what the Screening Brief Intervention and Referral Treatment is upon first mentioning in the introduction.
8. The authors should consider adding to their main research questions after mentioning the aim of the study at the end of the introduction.
9. Authors should also consider adding why the study was developed, why it is important, and what benefits will it have/add to research.

**Methods:**

Consider the following major and minor points for improvement:

10. Punctuation missing in the participant’s section.
11. Authors should include how the participants were recruited and via what means?
12. Authors should make the inclusion criteria clearer in the participant's section as well as add how many participants were included in the study.
13. What is the icebreaker question mentioned in the data collection section? Authors should add this and/or include examples.
14. Consider adding additional sections here to further explain the methods (it may be useful to add a semi-structured interview section, as well as a focus group section and explain what they are, how they were used in the study, etc.).
15. Authors should add a sentence or two to explain to readers what the grounded theorization approach mentioned is in the analysis section.
16. Authors should check for any grammatical errors, spelling, and punctuation throughout the methods section.

**Results:**

Consider the following major and minor points for improvement:

17. Authors switch between fully written out numbers and numerals. Check consistency throughout the paper.
18. The authors should consider including a table of the themes and subthemes to the results section to make these clearer for the reader.
19. Consider renaming some of the theme and subtheme headings to be more technically sound/scientific and less vague
20. Authors could make it clearer as to what information in the results section is from the semi-structured interviews and what is from the focus groups.
21. The authors should connect each theme back to the research questions and in the discussion.
22. Subtheme titled `The “bonus” of each profession`- authors should distinguish between using double and single quotation marks when directly quoting participant interviews and headings. Consider using single quotations in this title and in text (i.e., The ´bonus´ of each profession). Check for consistency throughout paper.
23. Consider adding to the *Debriefing* theme. This appears very short compared to the others.
24. Authors should check for any grammatical errors, spelling, and punctuation throughout the results section.

**Discussion:**

Consider the following major and minor points for improvement:

25. It is recommended that authors provide more information when linking their findings back to past research. Authors should consider doing this throughout the discussion section.
26. Authors should distinguish between using double and single quotation marks when directly quoting participant interviews and headings.
27. Authors should avoid directly quoting participants in their discussion section – this is for the results section. It is recommended that the discussion should be used merely to discuss the findings and link back to past studies.
28. Authors should check for any grammatical errors, spelling, and punctuation throughout the discussion section.
29. Authors should consider re-writing and keeping the strengths and weaknesses section short with a specific focus on the strengths, limitations, and future directions of the study (1-2 paragraphs). Authors include a lot of information that should be in the methods section of the paper. The authors should consider moving relevant method information earlier (i.e., how focus groups were arranged, took place, etc.), and remove repetition and other irrelevant information.
30. Figure 1 is blurry.

**Conclusion:**

Conclusion is to the point and easy to read!

Consider the following minor point for improvement:

31. The first sentence of the conclusion is identical to the first sentence of the discussion. Authors should consider re-writing to avoid repetition.
